# Antigenic Characterization of Neuraminidase of Influenza A/H7N9 Viruses Isolated in Different Years

**DOI:** 10.3390/ph15091127

**Published:** 2022-09-09

**Authors:** Yulia Desheva, Igor Losev, Nadezhda Petkova, Polina Kudar, Svetlana Donina, Andrey Mamontov, Chih-Hsuan Tsai, Yu-Chan Chao

**Affiliations:** 1 Virology Department, Federal State Budgetary Scientific Institution, Institute of Experimental Medicine, 197376 Saint Petersburg, Russia; 2 Institute of Molecular Biology, Academia Sinica, Taipei 115, Taiwan; 3 Department of Microbiology and Immunology, National Cheng Kung University, Tainan 701, Taiwan; 4 Department of Entomology, National Chung-Hsing University, Taichung 402, Taiwan; 5 Department of Entomology, National Taiwan University, Taipei 106, Taiwan

**Keywords:** influenza A/H7N9, neuraminidase, antibodies, live influenza vaccine, monoclonal antibodies

## Abstract

Influenza outbreaks caused by A/H7N9 viruses have occurred since 2013. After 2016, A/H7N9 influenza viruses underwent evolutionary changes. In this study, we examined the antigenic properties of influenza neuraminidase (NA) of A/H7N9 viruses as part of a live influenza vaccine (LAIV). It was shown that neuraminidase inhibiting (NI) antibodies obtained after A/Anhui/1/2013(H7N9)-based LAIV vaccination did not inhibit A/Hong Kong/125/2017(H7N9) NA and vice versa. The A/Hong Kong/125/2017(H7N9)-based LAIV elicited higher levels of NI antibodies compared to the A/Anhui/1/2013(H7N9)-based LAIV after two doses. Thelow degree of coincidence of the antibody response to hemagglutinin (HA) and NA after LAIV vaccination allows us to consider an enzyme-linked lectin assay (ELLA) as an additional measure for assessing the immunogenicity of influenza vaccines. In mice, N9-reactive monoclonal antibodies (mABs) for the A/environment/Shanghai/RL01/2013(H7N9) influenza virus partially protected against lung infection from the A/Guangdong/17SF003/2016 IDCDC-RG56N(H7N9) virus, thus showing the cross-protective properties of monoclonal antibodies against the drift variant.

## 1. Introduction

During the period from March 2013 to February 2015, 571 laboratory-confirmed cases of infection with the avian influenza A/H7N9 virus were registered in China, of which 212 (37.1%) were fatal [1]. Currently, avian influenza A/H7N9 virus strains are not capable of sustained transmission in the human population. Nevertheless, person-to-person transmission of the virus cannot be ruled out for at least 17 identified clusters consisting of 2–3 family members. According to experts, the average number of cases of secondary transmission in a susceptible population increased significantly as early as the second wave of the A/H7N9 outbreak in autumn 2013 [2].

By October 2017, the World Health Organization (WHO) had registered “six waves” of influenza A/H7N9 virus outbreaks. For the entire period from March 2013 to October 2017, there were 1567 laboratory-confirmed human cases, including 615 deaths [3]. Despite the use of a bivalent inactivated vaccine against H5 and H7 influenza in poultry in China [4], A/H7N9 viruses, which cause severe illness and death in humans, continue to circulate widely in some poultry populations and have pandemic potential, as most people probably do not have immunity to this subtype.

Genome analysis of human isolated A/H7N9 viruses showed adaptive evolution and convergent changes in eight viral genes, including the neuraminidase (NA) site (R289K). These substitutions are known to play a role in overcoming species barriers from birds to humans [5].

As a part of its pre-pandemic preparedness efforts, the WHO has developed a number of avian influenza A/H7N9 candidate vaccine viruses [https://cdn.who.int/media/docs/default-source/influenza/cvvs/zoonotic-cvv/h7n9-cvv/summary_a_h7n9_cvv_nh1920_20190220.pdf?sfvrsn=b9742e28_11], accessed on 21 October 2021. A number of A/H7N9 vaccines have been developed, including subunit, adjuvanted, and live influenza vaccines (LAIVs) [6,7,8,9]. The main criterion in the assessment of the immunogenicity of influenza vaccines is the antibody response to hemagglutinin (HA). At present, little is known about the antigenic properties of NA of A/H7N9 viruses as a vaccine antigen.

The aim of this study was to compare the antigenic properties of the NA of A/H7N9 viruses isolated before and after 2016. For these purposes, we studied the archival sera of volunteers obtained during clinical trials of two A/H7N9 LAIVs [8,9].

## 2. Results

### 2.1. Molecular Analysis of NA A/H7N9 Viruses

**Figure 1:**
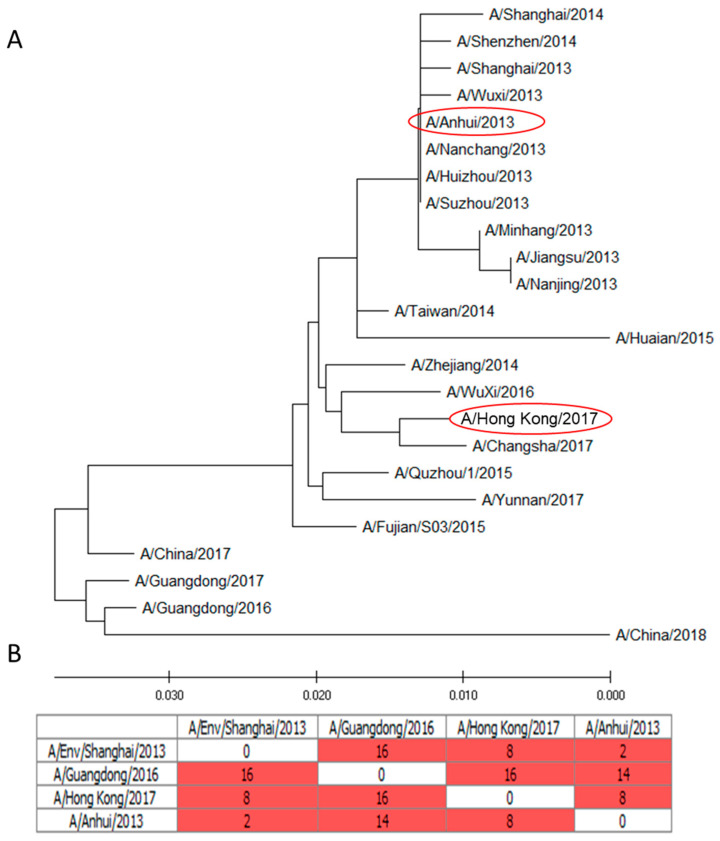
Evolutionary analysis of NA A/H7N9 viruses. (**A**) Phylogenetic tree of 24 amino acid sequences of influenza A/H7N9 viruses. The optimal tree with the sum of branch lengths = 0.15418388 is shown. The tree is drawn to scale, with branch lengths having the same units as those of the evolutionary distances used to infer the phylogenetic tree. The evolutionary distances were computed using the Poisson correction method [10] and are in the units of the number of amino acid substitutions per site. This analysis involved 41 amino acid sequences. All ambiguous positions were removed for each sequence pair (pairwise deletion option). There was a total of 465 positions in the final dataset. Vaccine strains are marked on philodendrogram. (**B**) Multiple sequence alignment distance matrix of the NA of viruses used in the study. The matrix demonstrates the number of amino acid substitutions between influenza virus strains.

The molecular genetic analysis showed that the A/H7N9 influenza viruses isolated from humans in 2017 and 2018 form a separate phylogenetic branch, in contrast to viruses isolated before 2016 (Figure 1).

The multiple alignment of the amino acid sequences of the N9 viruses showed that the NA of A/Anhui/1/2013(H7N9) and A/Hong Kong/125/2017(H7N9) LAIV viruses differed by 8 amino acid substitutions (Figure 1B): (I16T), (M72I), (Y166H), (V236A), (S242P), (I300V), (N322S), (A365T).

**Figure 2:**
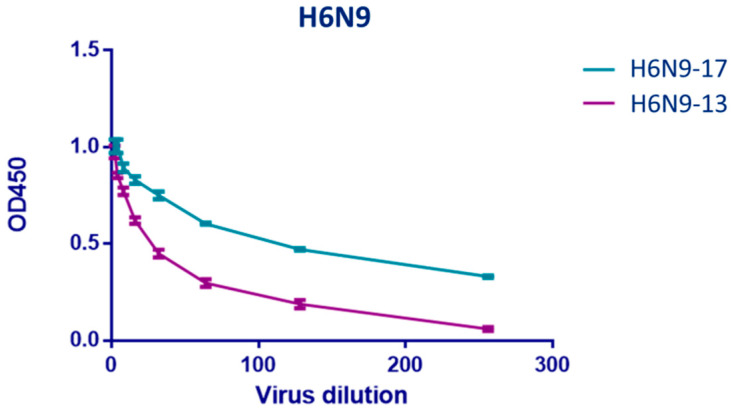
Dependence of the result of an enzymatic reaction on the dilution of viruses H6N9-13 and H6N9-17. The starting concentration of the influenza virus was 256 HAU.

We developed two A/H6N9 recombinant viruses in order to avoid non-specific reaction with antibodies to HA when analyzing neuraminidase inhibiting (NI) antibodies to A/H7N9 viruses. It was shown that the NA of A/Anhui/1/2013(H7N9) and A/Hong Kong/125/2017(H7N9) in recombinant A/H6N9 viruses differ in enzymatic activity with large molecular substrate fetuin (molecular weight, 50,000 [11]) (Figure 2). This follows from the differences in the optical density of wells containing NA desialyzed fetuin sites (Figure 2). The activity of the NA from the A/Hong Kong/125/2017(H7N9) virus was approximately two times higher compared to NA of A/Anhui/1/2013(H7N9). This circumstance was taken into account when selecting the working dose of viruses for determining NI antibodies.

### 2.2. Study of the Immunogenicity of LAIV Based on A/H7N9 Viruses Isolated in 2013 and 2017

**Figure 3:**
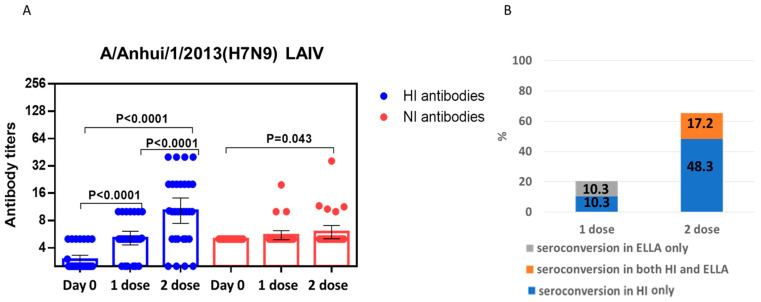
Serum antibodies after immunization with the first dose and the second dose of LAIV based on A/Anhui/1/2013(H7N9) (*n* = 29). (**A**) HI and NI antibody titers. Geometric means with 95% CI are presented. A four-fold increase in titer was considered a significant conversion for HI antibodies, and a two-fold increase in titer was considered a reliable conversion for NI antibodies. (**B**) Coincidence of HI and NI seroconversions (%) is presented.

First, the formation of hemagglutination inhibiting (HI) and NI antibodies was determined by an HI test and enzyme linked lectin assay (ELLA). It was shown that a significant increase in mean antibody titers of HI antibodies to the A/Anhui/1/2013(H7N9) virus was observed after the first dose, and the second dose 28 days later caused a statistically significant increase in antibodies compared with the first dose (Figure 3A). Mean NI antibody titers after the first dose did not statistically significantly differ from pre-vaccination levels, and a statistically significant increase in mean NI antibody titers was observed only after the second dose.

It was shown that after the first vaccination, there were no combined conversions of HI and NI antibodies. After the second vaccination, NI antibody seroconversions did not occur without HI antibody conversions, and the number of combined conversions was 17.2% (Figure 3B). Thus, the HA can be considered the dominant antigen in this case.

**Figure 4:**
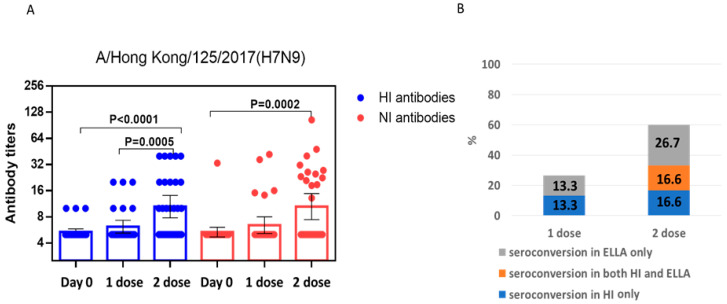
Serum antibodies after immunization with the first dose and the second dose of LAIV based on A/Hong Kong/125/2017(H7N9) (*n* = 30). (**A**) HI and NI antibody titers. Geometric means with 95% CI are presented. A four-fold increase in titer was considered a significant conversion for HI antibodies, and a two-fold increase in titer was considered a reliable conversion for NI antibodies. (**B**) Coincidence of HI and NI seroconversions (%) is presented.

Next, we studied the formation of serum antibodies after immunization with LAIV based on the A/Hong Kong/125/2017(H7N9) influenza virus. The results are presented in Figure 4. It was shown that the second dose of the A/Hong Kong/125/2017(H7N9)-based LAIV was more effective in relation to the production of NI antibodies (Figure 4A). At the same time, antibody titers after the second dose of /Hong Kong/125/2017(H7N9)-based LAIV were significantly higher than after the second dose of A/Anhui/1/2013(H7N9)-based LAIV (*p* = 0.01).

As in the case of A/Anhui/1/2013(H7N9)-based LAIV, after the first dose of A/Hong Kong/125/2017(H7N9)-based LAIV, there was no overlap in the conversions of HI and NI antibodies (Figure 4B). Conversely, after the second immunization, conversions of both HI and NI antibodies were observed both separately and simultaneously in the same sera. The number of combined conversions was 16.6%.

In general, immunization with A/H7N9 LAIV caused an increase in antibodies to HA and NA, on average, in more than 60% of vaccinated individuals. As was reported earlier, A/H7N9 LAIV also stimulated neutralizing antibodies and cellular immunity, so an increase in at least one parameter of the immune response was achieved in a much larger percentage of the vaccinated individuals [8,9]. The combined seroconversion of antibodies to HA and NA was not very common, which once again confirms the importance of these parameters as independent correlates of vaccination effectiveness.

**Figure 5:**
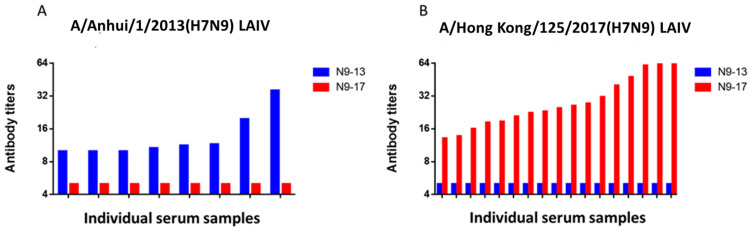
NI antibodies to antigenic variants of A/H7N9 after LAIV immunization. (**A**) Positive sera after A/Anhui/1/2013(H7N9)-based LAIV immunization (*n* = 8). (**B**) Positive sera after A/Hong Kong/125/2017(H7N9)-based LAIV immunization (*n* = 16).

Next, we tried to study whether there is a possibility of NI activity of antibodies acquired to the A/H7N9 virus isolated in 2013 against the A/H7N9 virus isolated in 2017 year and vice versa. To accomplish this, we took positive sera with different levels of antibodies to A/H7N9 viruses and tested them in the ELLA with a different antigenic variant. The data in Figure 5 show the complete absence of cross-reactions, since sera positive for one virus did not react with another and vice versa.

**Figure 6:**
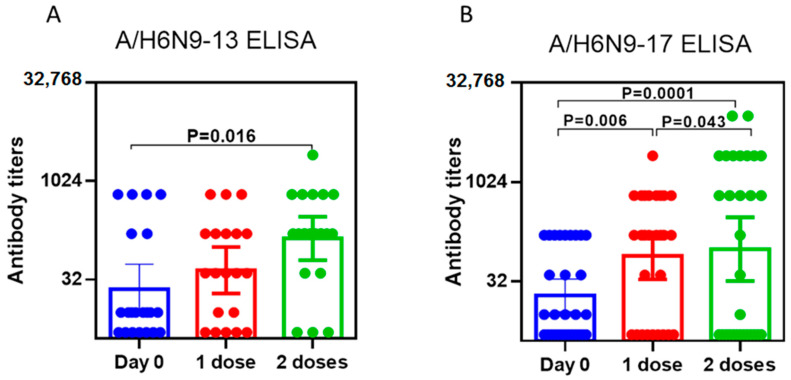
ELISA antibodies in the sera after A/H7N9 LAIV vaccination. (**A**) Immunization with A/Anhui/1/2013(H7N9)-based LAIV (*n* = 29). (**A**) Immunization with A/Anhui/1/2013(H7N9)-based LAIV (*n* = 29). Geometric means with 95% CI are presented (**B**) Immunization with A/Hong Kong/125/2017(H7N9)-based LAIV (*n* = 30). Geometric means with 95% CI are presented.

We also studied the increase in antibodies to A/H6N9-13 and A/H6N9-17 in the sera of vaccinated people using ELISA. The results are shown in Figure 6.

It was shown that after immunization with two doses of A/Hong Kong/125/2017(H7N9)-based LAIV, the levels of ELISA antibodies were higher than after immunization with two doses of A/Anhui/1/2013(H7N9)-based LAIV (Figure 6). A statistically significant rise in the mean antibody level was observed already after the first immunization with A/Hong Kong/125/2017(H7N9)-based LAIV.

Considering that ELISA antigens contained the H6 subtype of HA, the detected antibodies were not directed against HA of vaccine viruses, but were specific to the second surface antigen, NA, or, to a lesser extent, to other proteins, such as a nucleoprotein or a conserved HA stalk region [12,13,14].

### 2.3. Study of Monoclonal Antibodies (mABs) to NA Subtype N9

**Figure 7:**
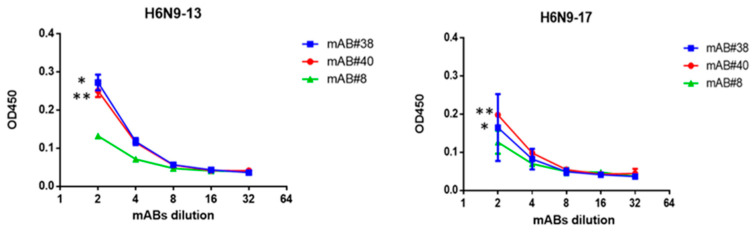
The binding curves of N9-specific mAB to A/H6N9-13 and A/H6N9-17 influenza viruses (20 HAU). Starting mAB concentration: mAB#8-0.47 mg/mL; mAB#38-0.5 mg/mL; mAB#40-0.5 mg/mL.* *p* < 0.05, Mann–Whitney U-test.** *p* < 0.05, Mann–Whitney U-test.

When comparing the average level of OD450 of serial dilutions of mABs in 96-well panels sensitized with 20 HAU of A/H6N9-13 or A/H6N9-17 viruses, a more pronounced affinity of mAB#38 and mAB#40 for the A/H7N9 virus isolated before 2016 compared to the virus isolated after 2016 was observed (Figure 7).

At the same time, mABs did not inhibit the enzymatic activity of N9 NA (data not shown).

**Figure 8:**
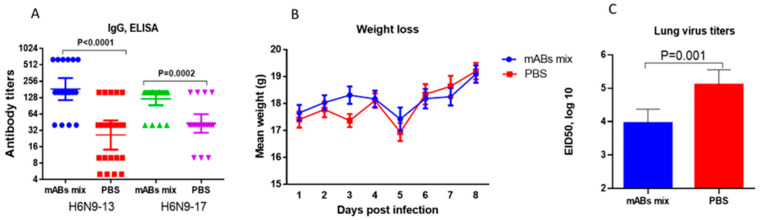
Results of mABs to N9 assessment in mice. (**A**) Serum IgG levels 5 h after immunization with mABs mix. The summary data of two experiments are presented (*n* = 20). (**B**) Changes in weight after intranasal infection with 300 MID_50_ of the A/Guangdong/17SF003/2016 IDCDC-RG56N(H7N9) influenza virus (*n* = 10). Mean values and standard error are presented. Data from one of two independent experiments are presented. (**C**) Reproduction of an infectious virus in the lungs on the fifth day after infection (*n* = 8). The data of two experiments are presented.

When mice were intraperitoneally injected with a mixture of mABs, N9-specific IgG was detected in the blood of the studied mice 5 h after passive immunization (Figure 8A). The mean levels of IgG to A/Anhui/1/2013(H7N9) NA were higher than to A/Hong Kong/125/2017(H7N9) NA, and the differences between vaccinated animals and PBS-treated mice were statistically significant. When intranasally introduced with 500 MID_50_ of the A/Guangdong/17SF003/2016 IDCDC-RG56N(H7N9) influenza virus, the infection was not lethal. After infection, the immunized mice lost weight relatively less compared to mock-immunized animals, but the differences were not statistically significant (Figure 8B). At the same time, a statistically significant decrease in the levels of infectious virus in the lungs was found among passively immunized animals (Figure 8C).

Thus, it was shown that through passive immunization, N9-reacive mABs were detected in the blood of immunized animals; mABs’ introduction did not significantly affect weight loss after infection with the antigenically distinct variant of A/H7N9, but led to a 10-fold decrease in the titers of the infecting virus in the lungs, and this decrease was statistically significant. These data suggest that the introduction of mABs had a weak therapeutic effect, which was expressed as a decrease in the infecting virus in the lungs.

## 3. Discussion

The pandemic potential of influenza viruses of the A/H7N9 subtype cannot be underestimated. The airborne transmission of human A/H7N9 isolates was demonstrated in a ferret model [15]. In addition, these viruses already have partially human-adapted phenotypes [16], and easily form NA inhibitor-resistant variants [17]. Despite the fact that there have been no outbreaks of A/H7N9 viruses since 2017 when vaccination with the inactivated H7 vaccine was started, this subtype remains prevalent in poultry in a number of Chinese provinces. At the same time, highly pathogenic avian A/H7N9 viruses begin to prevail over low pathogenic ones after the start of the use of an inactivated vaccine of this subtype. It has been shown that A/H7N9 viruses isolated from birds after 2019 form a separate phylogenetic branch that differs from A/H7N9 viruses isolated from 2016 to 2018. Thus, the evolution of A/H7N9 viruses is accelerated [18]. Much is known about the evolutionary variability of HA of A/H7N9 viruses [19]. NA also undergoes antigenic drift, but little is known about this. It is known that the NA of avian strains of A/H7N9 influenza was found to have a second catalytic site, which is not present in human strains. Due to those sites, they are able to bind erythrocytes, i.e., they have hemadsorption activity (HB). It was previously found that the HB site is formed by three NA loops: 367–372, 400–403, and 430–433 [20,21].

In our study, it was shown that human A/H7N9 viruses A/Anhui/1/2013(H7N9) and A/Hong Kong/125/2017(H7N9) have a similarity of 97% in NA amino acid sequence. At the same time, the enzymatic properties on A/Anhui/1/2013(H7N9) and A/Hong Kong/125/2017(H7N9) NA were different, in which the A/Hong Kong/125/2017(H7N9) virus NA demonstrated higher enzyme activity with high molecular substrate fetuin. This was also noticeable in terms of immunogenicity, since the titers of NI antibodies to the A/Hong Kong/125/2017(H7N9) influenza viruses were higher than those to A/Anhui/1/2013(H7N9) (Figure 3 and Figure 4). Thus, it is possible that the evolutionary variability of NA leads to an increase in enzymatic activity and immunogenicity. This was shown in our earlier work on the NA of A/H1N1pdm09 influenza viruses [22]. The current study shows that NI antibodies to A/H7N9 influenza viruses isolated in 2013 and 2017 not only differed in enzymatic activity, but also differed greatly in antigenic properties. Thus, NI antibodies formed to the A/H7N9 virus isolated in 2013 did not react at all with another A/H7N9 virus isolated in 2017 and vice versa, as shown in Figure 5.

It was noted that a significant increase in NI antibodies was obtained after the second immunization with LAIV based on both A/Anhui/1/2013(H7N9) and A/Hong Kong/125/2017(H7N9). The use of ELISA revealed thatlevels of antibodies to N9 was higher after the first and second vaccine doses of A/Hong Kong/125/2017(H7N9)-based LAIV compared to the A/Anhui/1/2013(H7N9)-based LAIV (Figure 6). It was previously shown that the enzyme activity of NA can serve as a measure of potential immunogenicity [23]. However, the immunogenicity of NA in the vaccine varies among the different strains of influenza viruses. For example, it has been shown that the immunogenicity of N1 and N2 antigens correlated with enzyme activity [24]. Thus, it might be worthwhile to study the enzymatic activity of the NA of influenza viruses considered as candidate vaccine strains.

In the event of a pandemic, the most effective way to fight influenza, as well as other viral infections, is vaccination. In this regard, it is important to develop, test, and deposit vaccines against emerging viruses. The created reserve will allow us, if necessary, to start production of vaccine preparations and the large-scale immunization of the population as soon as possible. It should be noted that the development of new vaccines cannot be accomplished without expanding the range of analyzed factors of the adaptive immune response in vaccinated people. Antibodies to NA—the second surface glycoprotein of the influenza virus—also have a protective effect, reducing the frequency of infection and the proportion of clinically pronounced forms in natural infection with drift and even shift variants of the influenza A virus [25].

One of the priorities of the WHO is to prepare for a new influenza pandemic, which includes the creation of collections of vaccine strains in order to start the production of a vaccine as soon as possible and immunize the population in the event of a pandemic threat A/H7N9 and assess the degree of susceptibility of the population to a potentially pandemic strain of influenza A/H7N9. The first stage of preparation involves a comprehensive, complete characterization of the immunogenicity and protective efficacy of vaccine preparations of a potentially pandemic subtype, including the use of methods for detecting a humoral immune response to influenza virus NA. The second stage includes the study of heterosubtypic anti-influenza immunity mediated by a number of factors, including NI antibodies.

Previously, it was shown that broadly neutralizing antibodies to seasonal influenza may be effective against pathogenic H7 strains and may contribute to the development of treatments against A/H7N9 strains [26]. Moreover, even NA-specific antibodies can protect against A/H7N9 infection [27]. In mouse study, we used a mixture of mABs to NA of A/environment/Shanghai/RL01/2013(H7N9). Using ELISA, it was shown that N9-specific mABs better bound to the NA of A/Anhui/1/2013(H7N9)13 influenza virus compared to the NA of A/Hong Kong/125/2017(H7N9) influenza virus (Figure 7). When administered parenterally, a mixture of N9-specific mABs was not only found in the blood of passively immunized animals (Figure 8A), but also partially protected against lung infection with the A/H7N9 virus isolated after 2016 (Figure 8B). Despite the fact that the introduction of these antibodies to mice had only a weak therapeutic effect, there was still a clear decrease in the infectious A/Guangdong/17SF003/2016 IDCDC-RG56N(H7N9) virus in the lungs. Thus, the cross-protective effect of mABs to N9 was shown.

## 4. Materials and Methods

### 4.1. Ethics Statement

In this study, we used archived blood serum samples from volunteers aged 18–49 taken as part of clinical trials in 2014 (ClinicalTrials.gov, Identifier: NCT02480101) [8] and in 2019 (ClinicalTrials.gov, Identifier: NCT03739229) [9]. Aliquots of serum samples taken from vaccinated individuals before vaccination, on day 28 after the first and second immunizations, were stored at −80 °C. The study was approved by the Local Ethics Committee at the FSBSI “IEM” (protocol dated (protocol dated 06 May 2020)). Upon receiving approval from the Ethics Committee, the sera were handed over to the researchers, none of whom had access to the personal data of patients (before analyzing the clinical data, the primary patient data were depersonalized and the researchers could not access them). As this is a retrospective study, informed consent was not required.

### 4.2. Viruses

The A/H7N9 LAIV vaccine strains were a 6:2 reassortant developed in the Institute of Experimental Medicine (St Petersburg, Russian Federation) based on A/Leningrad/134/17/57(H2N2) master donor strain and human A/Anhui/1/2013(H7N9) and A/Hong Kong/125/2017(H7N9) strains [8,9]. To assess NI antibodies, we developed two A/H6N9 viruses containing HA from the A/herring gull/Sarma/51c/2006(H6N1) strain. The H6N9-13 virus containing NA from A/Anhui/1/2013(H7N9) strain was prepared using classical genetic reassortment in chicken eggs (CE), as described elsewhere [28]. The H6N9-17 virus containing NA from A/Hong Kong/125/2017(H7N9) strain was prepared by means of standard plasmid-based genetic technique [29]. To assess hemagglutination-inhibition (HI) antibodies, we used vaccine strain A/17/Anhui/2013/61(H7N9) [8] and A/Hong Kong/125/2017 IDCDC-RG56B influenza virus, which was provided by Centers for the Diseases Control and Prevention, Atlanta, USA. The A/Guangdong/17SF003/2016 IDCDC-RG56N(H7N9) influenza virus which was used for mouse infection was provided by Centers for the Diseases Control and Prevention, Atlanta, USA. All viruses were propagated in the allantois cavity of 10-day developing chicken eggs (CE) supplied by “Sinyavino” poultry farm (Leningrad Region, Russia) at the 33 °C for 48 h, aliquoted, and stored at −70 °C.

### 4.3. Evolutionary Analysis of NA Amino Acid Sequences

Amino-acid sequences used in this study are available under the following accession numbers: AGQ88512, AGN69476, AGT41969, APR73187, AHZ60099, AGY41895, AGJ73505, AGL44440, AGR43209, AJJ91923, AIK26586, AJU15326, APR73167, AKI82234, MG214221, MF370248, MW142383, AIS16351, ISL249269, ARG43209, KY643848, ISL280902, MN170547, AID70635.

Multiple alignment was carried out using UGENE software from Unipro (Novosibirsk, Russia) [30]. The evolutionary history was inferred using the neighbor-joining method [31]. Evolutionary analyses were conducted in MEGA X [32].

### 4.4. Enzyme-Linked Lectin Assay (ELLA)

To assess NI antibodies in ELLA, we used two A/H6N9 viruses containing HA from the A/herring gull/Sarma/51c/2006 (H6N1) strain and NA from A/Anhui/1/2013(H7N9) or A/Hong Kong/125/2017(H7N9). The A/H6N9 viruses were concentrated and purified on a stepwise gradient of sucrose. Egg-propagated viruses were clarified by centrifugation at 3000 rpm for 20 min. The supernatants were transferred to ultracentrifuge vials (Nalgene, Thermo Fisher Scientific, Waltham, MA USA) and centrifuged at 17,000 rpm for 3 h in a Beckman coulter Optima™ L-100 XP Ultracentrifuge, Type 19 rotor (Beckman coulter, Brea, CA, USA). The supernatant was removed, and the pellet was resuspended in 2.0 mL of ice-cold calcium borate buffer (pH = 7.2). The resuspended virus was applied to a stepwise 30/60% sucrose gradient prepared in calcium borate buffer, followed by centrifugation at 20,000 rpm for 2.5 h (SW 40 Ti rotor). The virus-containing layer was taken and purified from sucrose in calcium-borate buffer by ultracentrifugation at 20,000 rpm for 2.5 h (SW 40 Ti rotor). The pellet was resuspended in 1.0 mL calcium borate buffer. Afterwards, 96-well plates (Greiner Bio-One, Kremsmünster, Austria) were coated with 50 µg/mL fetuin (Sigma-Aldrich, St. Louis, MO, USA) overnight at +4 °C. Next, 60 μL of each serum was heated at 56 °C for 30 min, serially diluted with phosphate-buffered saline containing bovine serum albumin (PBS-BSA), and then incubated with an equal volume of pre-diluted virus at a working dose of 64–128 hemagglutinating units (HAU) per 0.1 mL for 30 min at 37 °C. After incubation, 100 μL of the mixtures were applied to the fetuin-coated wells which were washed 3 times. After incubation for 1 h at 37 °C, the plates were washed, before assessing NA activity by incubating with peroxidase-labeled peanut lectin (2.5 μg/mL, Sigma-Aldrich, St. Louis, MO, USA) for 1 h at room temperature, followed by washing and addition of 100 μL of the peroxidase substrate 3,3′, 5,5′-tetramethylbenzidine (TMB). The reaction was stopped after 5 min by adding 100 μL of 1 N sulfuric acid. Optical density (OD) values were measured at 450 nm using a universal microplate reader (Elx800, Bio-Tek Instruments Inc., Winooski, VT, USA). Titers of serum antibodies against NA were determined as the reverse dilution of the sample with 50% inhibition of NA activity. A two-fold increase in NI antibody titer after vaccination was considered significant.

### 4.5. Hemagglutination Inhibition Test (HI)

HI test was performed using a 0.75% suspension of human erythrocytes (group “0”) in 96-well U-bottomed polymer plates as described previously [33]. The sera studied by us were treated with receptor-degrading enzyme (RDE) from *Vibrio cholerae* NA extract (Denka Seiken Co., Tokyo, Japan) and heated at 56 °C for 30 min. Each serum (in duplicates) was serially diluted starting from the initial dilution at 1:5 or 1:10 and mixed with 8 HAU of testing influenza virus. Antibody titers were expressed as the reciprocal of the highest serum dilution at which inhibition of agglutination was observed. A four-fold increase in HI antibody titer after vaccination was considered significant.

### 4.6. Enzyme-Linked Immunosorbent Assay (ELISA)

Serum IgG levels were determined using ELISA in 96-well ELISA plates (Sarstedt, Nümbrecht, Germany) coated with 20 HAU per 0.1 mL of the whole purified A/H6N9 viruses as previously described [34] in which the starting serum dilution was 1:10. HPR-labeled goat anti-human IgG (Sigma-Aldrich, St. Louis, MO, USA) was used as conjugate. The end-point ELISA titers were expressed as the highest dilution that yielded an optical density at 450 nm (OD450) greater than the mean OD450 plus 3 standard deviations of negative control wells.

### 4.7. Passive Immunization of Mice with Monoclonal Antibodies to N9

Anti-N9 NA antibodies were prepared at the Institute of Molecular Biology, Academia Sinica, Taipei, Taiwan. Specifically, cDNA sequence encoding the NA of A/environment/Shanghai/RL01/2013(H7N9) influenza virus was synthesized, and a recombinant baculovirus NA9-Bac displaying the NA protein was generated according to previous methods [34,35,36]. Inbred 6- to 8-week-old female BALB/c mice were intraperitoneally immunized with three shots of NA9-Bac (1 × 10^9^ plaque-forming units (PFU) per shot) at two-week intervals. Two weeks after the last immunization, splenocytes were harvested from mice, and monoclonal anti-N9 antibodies were produced by hybridoma technology. Three mABs with high binding affinity to N9 protein (i.e., NA9-Bac mAB#8, NA9-Bac mABB#38, and NA9-Bac mAB#40) were selected and amplified by the mouse ascites method. The final concentrations of these mABs are mAB#8-0.47 mg/mL; mAB#38-1 mg/mL; and mAB#40-1 mg/mL. All animal experiment procedures were approved by the Institutional Animal Care and Use Committee (IACUC) of Academia Sinica, Taiwan.

In the experiments of passive immunization of mice with NA9-Bac mABs, all animal procedures were carried out according to the “Rules of Laboratory Practice” (Russian Ministry of Health). The female 8–10-week-old CBA mice were purchased from the breeding laboratory (Rappolovo, Leningrad Region). The mice (20 per group) were lightly anesthetized with ether and immunized using a mix of mABs (15 μg of each per mouse in 0.1 mL). Animals in the control group were inoculated with PBS using the same route. Blood samples were obtained from the submandibular plexus 5 h after immunization. At the same time, mice were infected intranasally with 500 50% mouse infectious dose (MID_50_) of A/Guangdong/17SF003/2016 IDCDC-RG56N(H7N9) influenza virus. The animals were euthanized under ether anesthesia and cervical dislocation in order to determine the viral load in the lungs. To determine the viral titer in the lungs, the samples were homogenized in PBS containing 100 U/mL penicillin and 100 μg/mL streptomycin, and centrifuged for 10 min at 6000× *g*. To determine lung virus titers, the samples were titrated in CE starting from a dilution of 1:10. Virus titers were expressed as log10 50% embryo infective dose (EID_50_) as described previously [33]. The change in weight was observed within two weeks after the onset of primary viral infection.

Serum IgG levels in mouse sera were determined using ELISA as previously described with the difference being that HPR-labeled goat anti-mouse IgG (Sigma, St. Louis, MO, USA) was used as conjugate.

### 4.8. Statistical Analysis of Results

Data were analyzed using Statistica software, version 6.0 (StatSoft Inc., Tulsa, OK, USA) and graphics were generated using Prism 8 (GraphPad software, San Diego, CA, USA). Antibody levels were presented as geometric mean titers (GMT). For statistical analysis, antibody titers were expressed as reciprocal final dilutions of log2. Means and standard deviations of means (SD) were calculated to represent viral titers. Two dependent variables were compared using Wilcoxon matched pair test. Comparisons of two independent groups were performed using the nonparametric Mann–Whitney U-test. A *p* value < 0.05 was considered statistically significant.

## 5. Conclusions

It was shown that there was no cross interaction of NI antibodies to the A/Anhui/1/2013(H7N9) and A/Hong Kong/125/2017(H7N9) influenza viruses. The A/Hong Kong/125/2017(H7N9)-based LAIV elicited higher levels of NI antibodies compared to the A/Anhui/1/2013(H7N9)-based LAIV after the second dose. The revealed low degree of coincidence of the serum immune response to HA and NA after LAIV vaccination allows us to consider ELLA as an additional criterion for assessing the immunogenicity of influenza vaccines. In mice, mABs to NA of the 2013 A/H7N9 virus partially protected against lung infection with the A/H7N9 virus isolated after 2016. Thus, it can be assumed that the presence of NA-reactive antibodies that do not inhibit NA activity can still limit influenza infection.

## Data Availability

Data is contained within article.
